# Positive Effect of Large Birth Intervals on Early Childhood Hemoglobin Levels in Africa Is Limited to Girls: Cross-Sectional DHS Study

**DOI:** 10.1371/journal.pone.0131897

**Published:** 2015-06-29

**Authors:** Robel Afeworki, Jeroen Smits, Jules Tolboom, Andre van der Ven

**Affiliations:** 1 Nijmegen Institute for International Health, Radboud University Medical Centre, Nijmegen, The Netherlands; 2 Nijmegen Center for Economics, Institute for Management and Research, Radboud University, Nijmegen, The Netherlands; 3 Department of Paediatrics, Radboud University Medical Centre, Nijmegen, The Netherlands; London School of Hygiene and Tropical Medicine, UNITED KINGDOM

## Abstract

**Background:**

Short birth intervals are independently associated with increased risk of adverse maternal, perinatal, infant and child outcomes. Anemia in children, which is highly prevalent in Africa, is associated with an increased risk of morbidity and mortality. Birth spacing is advocated as a tool to reduce anemia in preschool African children, but the role of gender differences and contextual factors has been neglected. The present study aims to determine to what extent the length of preceding birth interval influences the hemoglobin levels of African preschool children in general, as well as for boys and girls separately, and which contextual factors thereby play a crucial role.

**Methods and Findings:**

This cross-sectional study uses data from Demographic and Health Surveys (DHS) conducted between 2003 and 2011 in 20 African countries. All preschool children aged 6–59 months with a valid hemoglobin measurement and a preceding birth interval of 7–72 months as well as their corresponding multigravida mothers aged 21–49 years were included in the study. Hemoglobin levels of children and mothers were measured in g/l, while birth intervals were calculated as months difference between consecutive births. Multivariate analyses were done to examine the relationship between length of preceding birth interval and child hemoglobin levels, adjusted for factors at the individual, household, community, district, and country level. A positive linear relationship was observed between birth interval and the 49,260 included children’s hemoglobin level, whereby age and sex of the child, hemoglobin level of the mother, household wealth, mother’s education and urbanization of place of residence also showed positive associations. In the interaction models, the effect of a month increase in birth interval is associated with an average increase of 0.025 g/l in hemoglobin level (P = 0.001) in girls, while for boys the effect was not significant. In addition, for girls, the effect of length of preceding birth interval was highest in young mothers and mothers with higher hemoglobin levels, while for boys, the highest effect was noticed for those living in more highly educated regions. Finally, significantly higher hemoglobin levels of girls compared to boys were observed at birth but with increasing age, the sex difference in hemoglobin level gradually becomes smaller.

**Conclusions:**

A longer birth interval has a modest positive effect on early childhood hemoglobin levels of girls, and this effect is strongest when their mothers are in their early twenties and have a high hemoglobin level. Remarkably, although the physiological iron requirement is higher for boys than girls, birth spacing has little influence on hemoglobin levels of preschool boys. We speculate that the preference for male offspring in large parts of Africa significantly influences nutritional patterns of African preschool boys and girls, and as such also determines the different effect of birth spacing. Finally, gender aspects should be considered in intervention programs that aim to improve anemia in African children.

## Introduction

Anemia is a major public health problem, affecting 1.62 billion people worldwide. The prevalence of anemia in low-income countries is around 43% in contrast to 9% in high-income countries [1]. Children and women are most commonly affected; the global anemia prevalence is 47% in children younger than 5 years, 42% in pregnant women, and 30% in non-pregnant women aged 15–49 years [2, 3]. The highest burden is observed in Sub-Saharan countries where 68% of preschool children and 57% of pregnant women have anemia [1]. Research indicates that pre-pubertal girls generally have higher hemoglobin levels than boys [4], but reliable data for African children are lacking.

The etiology of childhood anemia in Africa is multifactorial, including nutritional iron and folate deficiencies [5, 6], and parasitic diseases like malaria, hookworm and schistosomiasis [7, 8]. An integrated package of interventions is therefore recommended to address the multiple causes of anemia, that includes food fortification and family planning birth spacing as strategy [9]. Generally, birth spacing is known to influence different outcome measures for the mother, newborn and child, for instance the prevalence of stunting and underweight decreases as birth interval increases [10, 11]. Similarly, maternal depletion syndrome (MDP), that is characterized by a negative change in maternal nutritional status during the reproductive cycle and which is influenced by the length of birth interval, also impairs the health outcome of children [12, 13]. One may hypothesize that increasing the birth interval may result in less anemia as well, as the mother’s body will have more time to fully recover from giving birth and replenish stores of nutrients, including iron that may have been exhausted during pregnancy, delivery and lactation. Lactation anemia may be quite common under less privileged conditions as was for example shown in a study on anemia in breastfeeding Ethiopian women[14].

Delaying pregnancy until the mother’s current child reaches at least its second birthday increases the likelihood that the child gets adequate care during its first vulnerable years. It also may lengthen the lactating period, which is favorable as breast milk continues to be an important source of nutrition during the second year of life [15, 16].

Despite the general consensus about the positive effects of birth spacing, the optimal duration of the interpregnancy interval is not clear. Some studies suggest that a birth interval of 2–3 years reduces child mortality while on the other hand recent studies from the United States Agency for International Development (USAID) recommend a birth interval of 3–5 years [17, 18]. An interval larger than 5 years is generally not recommended because of the increased risk of neonatal mortality [10].

Socioeconomic status, lifestyle, stress, and adequacy of prenatal care, which are all associated with birth interval length, may also influence maternal, prenatal, infant, and child outcomes [19]. However, Conde et.al. provide evidence that adverse health outcomes associated with short interpregnancy intervals are not the result of sociodemographic, behavioral, or reproductive risk factors [20]. Research studies from low as well as high-income countries found the association between short interpregnancy intervals and adverse pregnancy and child outcomes to persist after controlling for many potential confounders (Conde-Agudelo et.al.2005; Stamilio et.al.2007; DaVanzo et al.2008; Rutstein 2008) [11, 20–22].

Although there is some circumstantial evidence suggesting a positive association, large-scale studies exploring the relation between birth spacing and anemia in pre-school children are still lacking, despite the circumstantial evidence suggesting a positive relation. The aim of the present study is therefore to determine to what extent the length of preceding birth interval influences the hemoglobin levels of African pre-school children in general, as well as for boys and girls separately and which contextual factors thereby play a crucial role.

## Methods

### Study population

This study used cross-sectional data derived from the Demographic and Health Surveys (DHS) [23, 24]. DHS are large, nationally representative household surveys, held in many low-income countries since the 1980’s, measuring indicators of population, health, and nutrition, with special emphasis on maternal and child health [25]. For each survey, non-overlapping area units (often enumeration areas) are randomly selected. These areas (called ‘clusters’ henceforth) are communities, villages or city quarters. In the selected clusters, all households are listed and a random sample of 20–25 households in urban areas and 25–30 households in rural areas is selected for the interview [24]. Each survey consists of a household interview, in which basic information is collected on all household members, and separate women’s and men’s surveys. In the women’s survey, all usual resident women aged 15 to 49 are invited for an oral interview. In this interview, information is obtained on socioeconomic, demographic, and health related issues. We included the most recent DHS survey available at time of our research for 20 African countries. Survey selection was based on availability of hemoglobin levels for women and children in the women’s survey.

Hemoglobin levels of children were available for children of up to 59 months. Within the 20 countries, 142 subnational regions (henceforth called ‘districts’), and 1999 clusters were distinguished. Information at district and cluster level was used to study effects of context factors. In all analyses, the household weights provided by DHS were used to obtain national representative samples. These weights were recoded to a mean of one, so that their application did not increase the sample sizes. For descriptive purposes, also national weights were constructed that made the sample representative of all countries together. To construct these national weights, the household weight factors were adjusted based on the World Bank population estimates for children aged 0–14 years within their respective country and year of survey [26]. Information on sample sizes, survey years and numbers of included children are presented in S1 Table.

Inclusion criteria were valid hemoglobin measurement, preceding birth interval of more than six months and less than six years and having a mother aged 21 and over. Mothers aged under 21 were excluded to avoid potential bias that might arise due to adverse outcomes of under-age pregnancies. A preceding birth interval of less than 7 months was considered implausible and of over six years as an indication of unusual household situation. The total number of children satisfying these criteria was 50,264. Of these children, 197 had to be removed because mothers BMI was missing, 147 because mother’s education was missing, 659 because the child’s age was missing and one because mother’s work was missing. The final study population therefore constituted of 49,260 children.

### Measurement

To measure hemoglobin levels, the blood of the women and children, obtained through a finger or heel prick, was tested for hemoglobin level. This testing was voluntary [23]. Consent to draw a droplet of blood was asked after reading a consent statement to the woman, parent, or responsible adult. The blood obtained was collected in a micro-cuvette. A battery operated portable Hemacue analyzer was used to measure hemoglobin concentration [24]. Hemoglobin level was measured in g/dL with a precision of 1 decimal. In this study, we use the unit g/L. To exclude outliers and input errors, the lowest and highest 0.5% of the hemoglobin levels was excluded from the analyses.

Preceding birth interval was expressed in months and defined as the period between the previous childbirth and the index child birth. The difference in months between two consecutive live births was considered a birth interval.

### Control factors

Control factors were included at the level of the children, the mother, the household and the context in which the household lived. Characteristics of the children that are associated with hemoglobin level include age (months), sex, birth order and twin status (singleton or multiple birth) [27–29]. Characteristics of the mother that have strong relationship with hemoglobin of child are age (years), hemoglobin level (g/l), body mass index (BMI, in kg/m2), currently breastfeeding (dummy variables for breastfeeding index child, breastfeeding other child, and no breastfeeding) [30, 31], status within the household (age difference with the partner), education (years received education), number of unions (not in a union, in first union, in second or later union), employment status (employed or not employed) [31, 32]. The term union included both married and cohabitating relationships. For women without a partner the mean was substituted on the age difference variable and a dummy variable was included to indicate women with a missing partner. At the household level, the DHS wealth index (five quintiles) was included [33].

Contextual factors were included at the cluster as well as at the district level. At cluster level, we included mean years of education of adults; preventive health measures (proportion of children having received vaccination against measles) and cluster altitude (in meters) [31, 34]. For clusters with missing altitude (flat countries), an altitude of 100m was substituted. At district level, we included level of development (percentage of households owning a television) and the availability of health facilities (proportion of last births delivered in a clinic/hospital). Descriptive information on the variables is presented in Table 1.

**Table 1:** Baseline characteristics of children (age 6–59 months) and their mothers (age 21–49 years) from 20 African countries (DHS database).

Characteristic	Category	Sample
		(N = 49,260)
**Preceding birth interval (months)**, mean (SD)	N/A	34 (13.2)
**Age of child (months)**, mean (SD)	N/A	32.1 (15.5)
**Sex**	Male (%)	50.5
	Female (%)	49.5
**Birth order**, mean (SD)	N/A	4.6 (2.3)
**Hemoglobin of child (g/l)**, mean (SD)	N/A	106.7 (17.2)
**Maternal Hemoglobin (g/l)**, mean (SD)	N/A	124.9 (18.2)
**Age of mother (years)**, mean (SD)	N/A	30.9 (6.1)
**BMI of mother (Kg/m2)**, mean (SD)	N/A	22.4 (4.3)
**Age difference between mother and partner (years)**,	N/A	7.8 (7.1)
mean (SD)		
**Mother’s education (years completed)**	N/A	3.5 (4)
**Twins in family** (%)	N/A	1.7
**Number of unions** (%)	Not in a union	6.2
	First union	78.9
	Second or later union	14.9
**Employed mother** (%)	N/A	62.2
**Breastfeeding mother** (%)	No breastfeeding	38.7
	Index child breastfeeding	35.1
	Other child breastfeeding	26.2
**Residence** (%)	Rural	79.3
	Urban	20.7
**Wealth index** (%)	Poorest	23.6
	Poorer	22.2
	Middle	21.9
	Richer	19.1
	Richest	13.2
**Altitude**, mean (SD)	N/A	936 (745)

### Statistical analysis

Bivariate and multivariate regression analyses were performed to determine the associations between the selected independent variables and the children’s hemoglobin level. All regression models contained fixed-effects dummy variables at the district level, with separate dummy variables for the urban and rural parts of the districts, to control for clustering and achieve complete control for direct effects of confounders at those levels. Given that all variation at the district level was controlled by these dummy variables, no direct effects of the context factors at the district level could be estimated. These context factors are therefore only used in the interaction analysis. We tested for nonlinearities by including quadratic terms for all interval variables, but only for the altitude variable the quadratic effect was significant. For altitude, this term was therefore included in the models.

Interaction analysis was used to examine the influence of characteristics of the children, their mothers, the households and the context on the strength of the preceding birth interval effect. We tested for significant interactions between the birth interval effect and the children’s age, birth order, their mother’s age, mother’s hemoglobin level, mother’s BMI, and mother’s age difference with her partner, household wealth, cluster educational level, district level of development and availability of health facilities. To adjust the significance level for multiple interaction testing, the Bonferroni correction was applied [35]. Given that nine interactions were tested, the critical P-value was reduced to 0.05/9 = 0.0056. Because the effect of preceding birth interval differed significantly between boys and girls, separate interaction analyses were performed for boys and girls.

## Results

**Fig 1:**
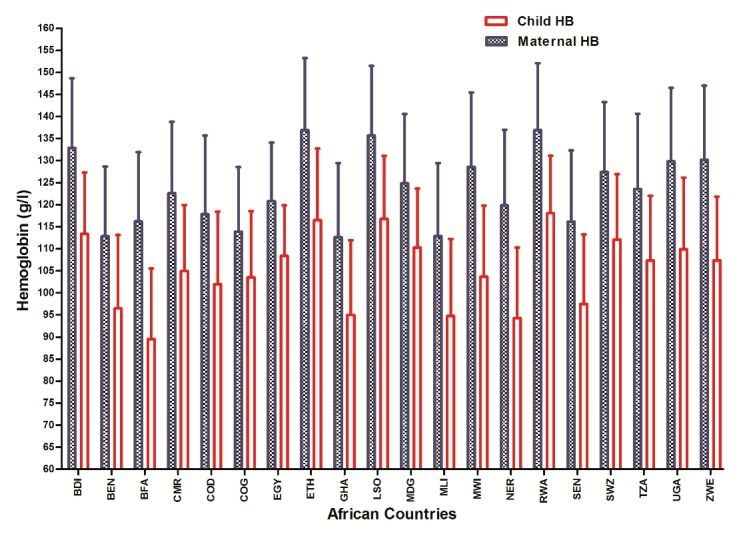
Mean hemoglobin levels of mothers and children in 20 African countries.

The baseline characteristics of children and mothers are described in Table 1. The hemoglobin levels of children and mothers for the 20 countries, that were normally distributed, are presented in Fig 1 The mean hemoglobin value for the mothers (125 ± 18.2 g/l) was significantly higher compared to that of the children (107± 18.2 g/l). High mean hemoglobin levels are observed in mothers and children living in mountainous countries, such as Ethiopia, Burundi, Rwanda, and Lesotho. Low mean hemoglobin levels are noticed among residents from the low altitude regions in West Africa including Benin, Burkina Faso, Ghana, Mali and Niger.

Coefficients of the bivariate regression analyses are presented in the second column of Table 2. The effect of the preceding birth interval variable on the index child’s hemoglobin level was positive but not significant. Girls are found to have a significant higher hemoglobin level than boys. All other variables, except being part of a multiple birth were significantly associated with the child’s hemoglobin level.

**Table 2:** Bivariate and Multivariate analyses for hemoglobin level of African children (age 6–59 months, n = 49,260) from 20 African countries (DHS database).

Variable	Bivariate Model	Multivariate Model
	Coefficient (SE)	Coefficient (SE)
Intercept	-	69.603 (0.954)**
**Preceding birth interval, months**	0.007 (0.005)	0.015 (0.005)**
**Children Factors**		
Sex is female (reference = male)	0.872 (0.134)**	0.847 (0.127)**
Age of child, months	0.232 (0.004)**	0.207 (0.006)**
Birth order	0.118 (0.03)**	0.000 (0.047)
**Household factors**		
Maternal Hemoglobin, g/l	0.147 (0.004)**	0.139 (0.004)**
Age of mother, years	0.181 (0.011)**	0.071 (0.018)**
BMI of mother, Kg/m^2^	0.003 (0.000)**	0.001 (0.000)**
Breastfeeding mother		
- Not breastfeeding and index child	Reference group	Reference group
- Breastfeeding and index child	-5.317 (0.157)**	-1.253 (0.206)**
- Breastfeeding mother and other child	0.638 (0.170)**	-0.390 (0.169)*
Multiple birth (Ref = Singleton)	0.003 (0.520)	-0.404 (0.497)
Wealth index	1.140 (0.062)**	0.751 (0.062) **
Age difference mother and partner, years	-0.038 (0.010)**	-0.028 (0.010)**
Education year of mother, years	0.225 (0.022)**	0.119 (0.022)**
Number of unions		
- First union	Reference group	Reference group
- Not in a union	-0.398 (0.282)	-0.452 (0.269)
- Second or later union	-0.403 (0.195)*	-0.303 (0.188)
Employed mother (Ref = no)	0.905 (0.166)**	0.295 (0.159)
**Contextual Factors**		
Altitude	0.08 (0.001)**	0.006 (0.001)**
Altitude^2^	-0.000 (0.000)**	-0.000 (0.000)**

* P<0.05

** P<0.01

BMI = Body Mass Index

The outcomes of the multivariate regression analysis are shown in the third column of Table 2. In this analysis, the effect of the preceding birth interval variable is significantly positive. The coefficient of this variable indicates that for every increase of one month of the preceding birth interval there is a gain of 0.015 g/l hemoglobin level.

The results again show that the hemoglobin level of girls is significantly higher than that of boys. We also observe that mother’s age, maternal BMI, maternal hemoglobin level, maternal educational level and her age difference with her partner all have significant positive effects on hemoglobin level of the index children. Household wealth has also a strong effect. Children living on higher altitude had significantly higher hemoglobin levels but the effect is nonlinear, with the effect starting to stabilize when the residence of the child was above 4,000 meters.

To analyze whether the effect of length of preceding birth interval affected hemoglobin levels of boys and girls differently, an interaction term was added to the multivariate model. This term turned out to be significant and positive (effect size 0.023 P = 0.019), which means that the preceding birth interval effect was substantially stronger for girls than for boys. Separate multivariate analyses for boys and girls made clear that the effect was only significantly present in girls (effect size 0.021, P = 0.003) and not in boys (effect size 0.004, P = 0.579).

### The role of the context

To analyze whether the effect of length of preceding birth interval depended on the circumstances in which the children were living, interactions between this effect and the other variables in the model were studied. Given the substantial sex difference in the birth interval effect, the interaction analysis was performed separately for boys and girls. Because little knowledge of such interactions was available, this analysis is largely explorative in nature. We tested all interactions between preceding birth interval and the major other variables in the model (children’s age, mother’s age, mother’s hemoglobin level, mother’s BMI, mother’s age difference with her partner, household wealth, cluster educational level, district level of development and availability of health facilities) and added the significant interactions to the model.

Table 3 presents the outcomes of this analysis. In the interaction models, the effect of a month increase in birth interval is associated with an average increase of 0.025 g/l in hemoglobin level (P = 0.001) in girls and of -0.000 (P = 0.948) in boys. After applying the Bonferroni correction, in girls the birth interval effect was significantly influenced by two factors: age of the mother (effect size -0.004, P = 0.001) and hemoglobin level of the mother (effect size 0.001, P<0.001). The interaction effect of age of the mother was negative, thus indicating that for daughters of young mothers a longer birth interval is particularly important. The interaction effect of the mother’s hemoglobin level was positive. This suggests that a longer birth interval helps girls in making full use of available nutritional resources. For boys, interactions with age of the mother and with district educational level were significantly positive at the P<0.05 level. However, only the interaction with district educational level remained intact after applying the Bonferroni correction (effect size 0.001, P<0.001). It indicates that boys are better able to profit of a longer birth interval in regions where women are more highly educated.

**Table 3:** Summary of separate interaction models for boys and girls.

	Coefficient (SE)	
Variable	Boys	Girls
Intercept	98.175 (1.039)**	95.854 (1.059)**
**Preceding birth interval, months**	-0.000 (0.008)	0.025 (0.007)**
**Preceding birth interval, months** * **Age of mother**	0.003 (0.001)*	-0.004 (0.001)^#^
**Preceding birth interval, months** * **Hb of mother**		0.001 (0.000)^#^
**Preceding birth interval, months** * **Mean education**	0.001 (0.000)^#^	
**level of mothers in region**		

Multivariate models for the relationship of hemoglobin level of children (age 6–59 months, n = 49,260) from 20 African countries (DHS database) and their preceding birth interval adjusted for all predictors.

* P<0.05

** P,0.01

^#^ P<0.0056 (Bonferroni correction)

Hb = Hemoglobin level

**Fig 2:**
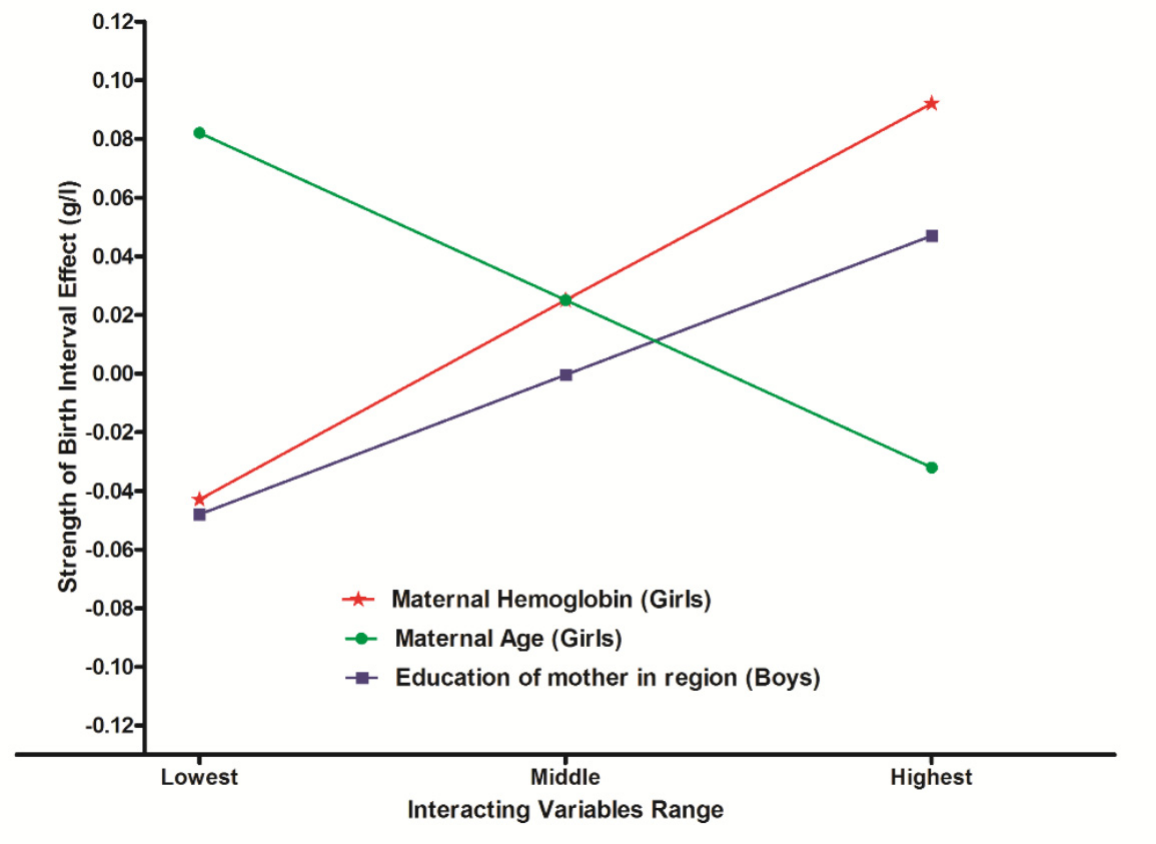
Variation of the preceding birth interval effect for girls according to maternal age (21–49) and maternal HB level (55–166 g/l) and of the preceding birth interval effect for boys according to average education of mothers in the district.

The interaction effects are visualized in Fig 2 The increase in hemoglobin level per month increase in birth interval is for girls with a young mother or girls with a mother with a high hemoglobin level about 0.12 g/l higher than for girls with an old mother or a mother with a low hemoglobin level. This is a difference of about 1.4 g/l for a birth interval increase of a year. For boys the effect of a month increase in birth interval was in areas with a high maternal educational level about 0.08 g/l higher than in areas with a low maternal educational level. This is a difference of about 1 g/l for an increase of a year.

## Discussion

Several important conclusions can be drawn from the present study with regard to the birth interval effect. First, we found that the length of the preceding birth interval has a modest positive effect on hemoglobin levels of African preschool girls, but not of that of boys. Second, having a long preceding birth interval is particularly important for African girls with a young mother. Third, girls were also found to profit more if their mother has a higher hemoglobin level. Fourth, boys were found to profit from a longer birth interval only under specific circumstances, namely if they lived in an area where the educational level of mothers is high.

Positive effects of increasing birth intervals on health outcomes of children and their mothers have been noticed in several studies [10, 11, 15, 36], whereby those from Condelo et.al. [17] and Rutstein et.al. [11] suggest that the optimal birth interval lies between 36–60 months. The present study is unique in using hemoglobin as outcome parameter and studying its effects in gender differences. Using hemoglobin level as outcome is important, because it is sensitive indicator of well being or general health status. As anemia is highly prevalent in African mothers and children, this indicates an urgent need for effective interventions [1, 2, 9].

The focus on gender differences turned out to be important too. When studying differences between boys and girls, while controlling for confounding factors, a significant effect of longer birth interval was particularly found for girls but turned out to be insignificant for boys. The positive effect observed in girls was in the order of 0.025 g/l for a one month increase in the birth interval, or 0.3 g/l for an increase of a year. In addition, we found that the highest hemoglobin increments were noticed in girls with young mothers and in girls with mothers with a high hemoglobin levels. Compared to girls with the oldest mothers in our data (late forties) and the mothers with the lowest hemoglobin levels (under 60), for those with the youngest mothers (early twenties) and with the mothers with the highest hemoglobin levels (over 160) the effect of a year increase in birth interval is associated with an approximate increase of 1.4 g/l in hemoglobin level. Given that the effects are additive, for girls with a young mother with a high hemoglobin level the birth interval effect is in the order of 2.8 g/l higher than for those with an old mother with a low hemoglobin level.

We did not find a significant overall effect of birth interval on hemoglobin level among boys. However, the interaction analysis made clear that in areas where mothers have relatively high educational levels, boys profit more from a longer birth interval than in areas where mothers have relatively low educational levels. This difference is in the order of 1 g/l for an increase of a year.

Childhood anemia is very prevalent in Sub-Sahara Africa but gender differences are thereby rarely considered. Our results suggest that increasing hemoglobin levels in mothers may positively affect hemoglobin levels in their daughters. Furthermore, it seems important to target the interventions especially to young mothers, as their female offspring profits most from longer birth intervals.

Our data were derived from the DHS, including 49,260 children in 20 African countries whereby many biological, maternal, socioeconomic and contextual factors were collected as well. Because of the large sample size, internal validity was secured and variance of estimates reduced. Adjustment for covariates was performed using multivariate analysis with country and region fixed effects. In these analyses, it was noticed that biological, maternal, socioeconomic, environmental and contextual factors were related to hemoglobin levels of African preschool children and that most of the factors affect hemoglobin levels of African boys and girls rather similarly.

Apart from the separate effect of the various factors, a common denominator may play a significant role in the different sex response to birth spacing. We hypothesize that the preference for male offspring in many African countries leads to more maternal investments in boys compared to girls, making them less susceptible for the effect of birth spacing. Studies from Ethiopia (Quisumbing,2003) [37] and Peru (Gerlter and Gleewe,1992) [38] indeed showed that larger investments in boys are made, while findings from India show that girls are disadvantaged with less investment in health inputs and outcomes [39].The difference in investments for boys compared to girls may be expressed through several socioeconomic and contextual factors. Studies from Africa show that birth spacing is shorter after the delivery of a girl than of a boy [40, 41] and in young compared to older mothers [41, 42]. The first factor may also have to do with sex preference and investment in boys while the age effect of mothers may relate difference in fecundity rate and timing of family building responsibilities [42].

The maternal depletion syndrome could be another factor that contributes to hemoglobin levels of their offspring. Maternal depletion syndrome is characterized by a deterioration of the maternal nutritional status during the reproductive cycle, especially when the period of depletion is long while the time for repletion is short and the mother has a marginally inadequate food intake [13, 15]. For instance, inadequate iron and folic acid intake during the reproductive period affect the maternal status for these nutrients during the interpregnancy interval and iron and folic acid deficiency has been associated with preterm births and fetal growth retardation.[43]. Lower fecundity or longer birth intervals may provide mothers with an opportunity window to replenish iron loss from a previous pregnancy and delivery and sustain physiological recuperation for the following one. Similarly, the offspring’s hemoglobin level may gain from a longer preceding birth interval as it depends on maternal iron status, beside other factors such as gestational age, birth weight and timing of umbilical cord clamping [44].

Given that no significant association between preceding birth interval and the children’s age was found, our study also indicates that the positive effects of a longer birth interval persist across the first 6 years of life. This may be explained by the fact that the age difference between the two succeeding children remains, which may create the opportunities for more maternal investments over the whole period. In addition, also biological factors, like epigenetic changes may contribute to the constant positive effect of a longer birth interval. The clinical relevance of our finding is difficult to give, as the effects on hemoglobin levels are only modest and mostly seen in girls that have already a greater chance of survival while boys are biologically weaker and more susceptible to disease and premature death [45, 46].

Apart from the birth interval effect, our analyses made clear that hemoglobin levels of African children steadily increase during the first 59 months of life (effect size 0.207, P<0.001) and that hemoglobin levels of girls were significantly higher than those of boys (effect size 0.847, P<0.001). An additional test for sex differences in the age effect revealed a significant interaction (effect size 0.045, P<0.001), whereby the increment with age is significantly stronger in boys (effect size 0.229, P<0.001) than in girls (effect size 0.183, P<0.001). Hence, with increasing age, the sex difference in hemoglobin level becomes smaller. In S1 Fig this sex difference in the age effect is visualized.

Various other studies have also reported higher hemoglobin levels of girls [27, 29]. Furthermore, different studies have reported higher rates of anemia and lower iron status in male compared to female infants, which were attributed to sex-related differences in iron requirement [39, 40, 42, 44]. Wieringa et al. showed in a study from South East Asia that boys have a higher risk to be anemic and to have iron deficiency anemia because of higher iron requirements while both girls and boys achieved similar hemoglobin concentrations after iron supplementation, indicating no reason to surmise physiological sex differences in hemoglobin set-points [47]. Also, a recent study indicated important sex differences in iron regulating hormone hepcidin levels in Kenyan children with anemia and iron deficiency, indicating that iron regulation may be different in boys compared to girls [48]. Apart from the direct effect of iron and iron regulating hormones, erythropoiesis is also influenced by other parameters that are gender dependent such as growth hormone (GH) and insulin-like growth factor (IGF-1). Indeed lower IGF-1 levels have been found in African boys [49].

Our study is limited by the lack of information on major co-morbidities that affect hemoglobin status, such as malaria, tuberculosis and Human Immunodeficiency Virus (HIV) infections as well as non-communicable diseases, like nutritional deficiencies, including those for folate, vitamin B12 and vitamin A. The same applies to inherited or acquired disorders that affect hemoglobin synthesis, red blood cell production or red blood cell survival. The overestimation effect of miscarriage and underestimation effect of stillbirth in the length of birth interval cannot be discussed upon due to restricted information of maternal pregnancy in DHS data. Further, the study uses a cross-sectional design, which prevents the inference of cause and effect relationship.

In conclusion, increasing birth interval has a moderate positive effect on hemoglobin levels of preschool African girls. Remarkably, although iron requirement of boys seem higher than girls, birth spacing did not influence hemoglobin levels of preschool boys. The preference for male offspring in large parts of Africa may not only significantly contribute to the sex differences in hemoglobin levels but may also determine the different effect of birth spacing on hemoglobin levels of African boys and girls. Finally, gender aspects should be considered in interventions programs that aim to improve anemia in African children.

## Supporting Information

S1 Fig Hemoglobin levels (in g/l) of boys and girls by age of the child (in months). (TIF)

S1 Table List of 20 African countries, year of survey, number and percentage of children included in the cross-sectional study. (DOC)
