# Supplementary figures and images for: Positive Effect of Large Birth Intervals on Early Childhood Hemoglobin Levels in Africa Is Limited to Girls: Cross-Sectional DHS Study
Source: PLoS One. 2015 Jun 29;10(6):e0131897. doi: 10.1371/journal.pone.0131897 (PMC4488302; doi:10.1371/journal.pone.0131897)

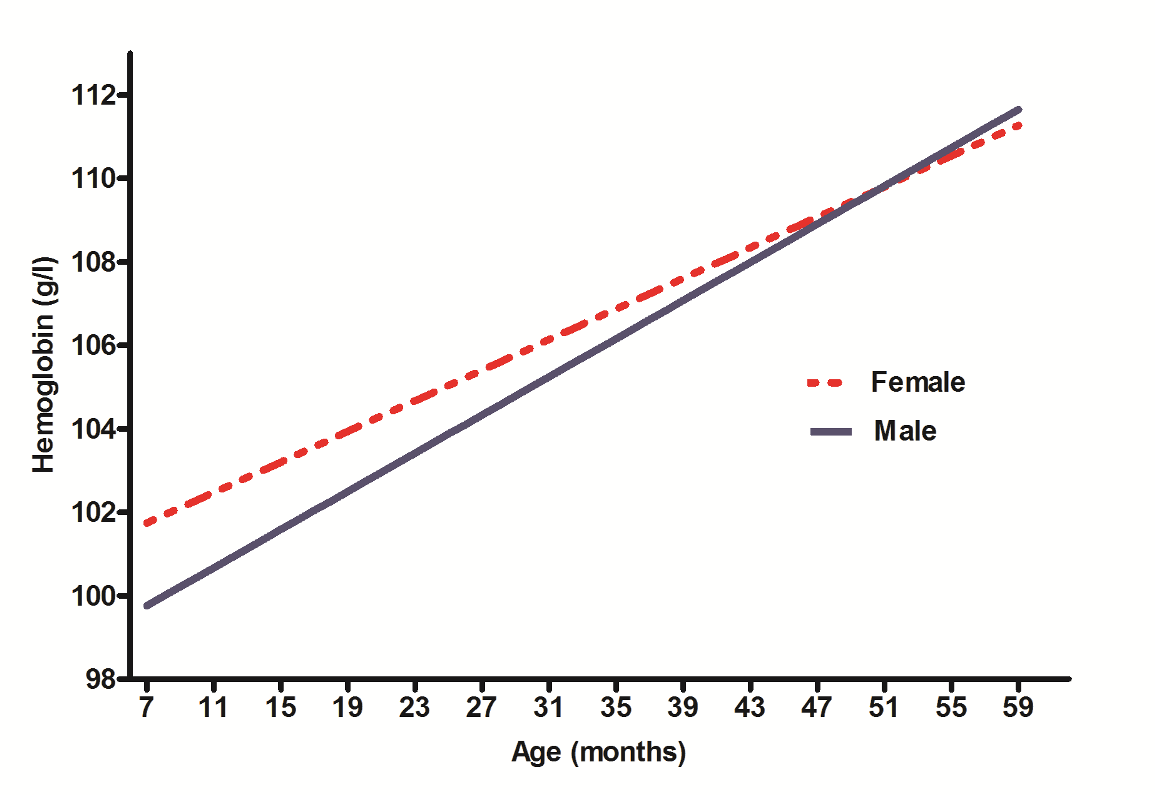

Supplement: S1 Fig — (TIF) [file pone.0131897.s001.tif]
