# Supplementary material for: Positive Effect of Large Birth Intervals on Early Childhood Hemoglobin Levels in Africa Is Limited to Girls: Cross-Sectional DHS Study
Source: PLoS One. 2015 Jun 29;10(6):e0131897. doi: 10.1371/journal.pone.0131897 (PMC4488302; doi:10.1371/journal.pone.0131897)
Supplement: S1 Table — (DOC) [file pone.0131897.s002.doc]

Table S1. List of 20 African countries, year of survey, number and percentage of children included in the cross-sectional study.

| **Country** | **Year of survey** | **Number of children** | **Percentage of** |
| --- | --- | --- | --- |
|  |  | **(Sample size)** | **Pre-school children** |
| Burundi | 2010 | 1,051 | 2.1 |
| Benin | 2006 | 816 | 1.7 |
| Burkina Faso | 2003 | 1,373 | 2.8 |
| Cameroon | 2011 | 2,170 | 4.4 |
| Congo-DR | 2007 | 7,047 | 14.3 |
| Congo Brazzaville | 2005 | 292 | 0.6 |
| Egypt | 2008 | 4,944 | 10.0 |
| Ethiopia | 2011 | 9,725 | 19.7 |
| Ghana | 2008 | 1,934 | 3.9 |
| Lesotho | 2010 | 117 | 0.2 |
| Madagascar | 2009 | 2,078 | 4.2 |
| Mali | 2006 | 1,308 | 2.7 |
| Malawi | 2010 | 1,778 | 3.6 |
| Niger | 2006 | 1,668 | 3.4 |
| Rwanda | 2010 | 1,234 | 2.5 |
| Senegal | 2011 | 1,196 | 2.4 |
| Swaziland | 2006 | 97 | 0.2 |
| Tanzania | 2010 | 5,129 | 10.4 |
| Uganda | 2011 | 4,447 | 9.0 |
| Zimbabwe | 2011 | 854 | 1.7 |
| **Total** |  | **49,260** | **100** |
|  |  |  |  |
